# Supplementary material for: An Exploratory Study of Refining TNM-8 M1 Categories and Prognostic Subgroups Using Plasma EBV DNA for Previously Untreated De Novo Metastatic Nasopharyngeal Carcinoma
Source: Cancers (Basel). 2022 Apr 11;14(8):1923. doi: 10.3390/cancers14081923 (PMC9031957; doi:10.3390/cancers14081923)
Supplement: Supplementary file 1 [file cancers-14-01923-s001.zip › cancers-1641661-supplementary.pdf]

cancers

MDPI

Article

An Exploratory Study of Refining TNM-8 M1 Categories and Prognostic Subgroups Using Plasma EBV DNA for Previously Untreated De Novo Metastatic Nasopharyngeal Carcinoma

Sik-Kwan Chan, Brian O'Sullivan, Shao Hui Huang, Tin-Ching Chau, Ka-On Lam, Sum-Yin Chan, Chi-Chung Tong, Varut Vardhanabhuti, Dora Lai-Wan Kwong, Chor-Yi Ng, To-Wai Leung, Mai-Yee Luk, Anne Wing-Mui Lee, Horace Cheuk-Wai Choi and Victor Ho-Fun Lee

Supplementary Methods

Pre-treatment Investigations and Details of Treatment and Survival Follow-up

(I) PET-CT Scan Protocol

Whole body [18F]fluorodeoxyglucose (18F-FDG) PET-CT was performed with a combined PET-CT scanner (Discovery VCT, 64 multislice spiral CT; GE Healthcare Bio-Sciences Corp) using a standardized protocol. All patients were immobilized in a supine treatment position by a custom-made thermoplastic head and neck cast before scanning. The scanning range was from the vertex of brain to the upper thigh. After six hours of fasting, 220 to 370 MBq [4.8 MBq/kilogram (kg)] of body weight-adjusted 18F-FDG was administered intravenously. After a 60-minute uptake time, whole-body emission PET scans were obtained with seven bed positions. Attenuation-corrected PET images with CT data were reconstructed with an ordered-subset expectation maximization iterative reconstruction algorithm (14 subsets and two iterations) and fused with CT images (Advanced Workstation 4·3; GE Healthcare Bio-Sciences). The CT imaging parameters were as follows: 120kVp; 200 to 400 mA; 0.5 second per CT rotation; pitch 0.984:1; and 2.5 mm intervals, with or without 60 to 100 ml (1.5 ml/kg of body weight) intravenous contrast medium.

(II) MRI Techniques

MRI scanning was performed on the following day after PET-CT scanning with a 3.0-T MR scanner (Achieva 3·0T, Philips Healthcare, Best, The Netherlands), utilizing a 16-channel neurovascular coil. Image acquisition was made in axial plane from suprasellar region cranially to lung apices caudally. Four standard sequences were performed: a) axial T1-weighted turbo spin echo (TSE) [repetition time/echo time (TR/TE) = 454/9·2 milliseconds (ms); turbo factor = 3; FOV = 230 × 230 millimeter (mm); matrix = 672 × 672; slice number = 32; slice thickness = 3 mm; intersection gap = 0.3 mm]; b) axial T2-weighted short TI inversion recovery (STIR) [TR/TE = 4644/60 ms; field-of-view [FOV] = 230 × 230 mm; matrix = 672 × 672; slice number = 32; slice thickness = 3 mm; intersection gap = 0·3 mm]; c) coronal T2-weighted STIR [TR/TE = 4644/60 ms; FOV = 230 × 230 mm; matrix = 480 × 480; slice number = 32; slice thickness = 3 mm; intersection gap = 0.3 mm]; d) 3D T1-weighted turbo-field- echo (TFE) post-contrast scan [TR/TE = 4.8/2.4 ms; flip angle = 100; FOV = 230 × 230 mm; matrix = 640 × 640; slice number = 319; slice thickness = 0.72 mm]. Intravenous bolus injection of 0.1 mmol/kg of body weight gadopentetate dimeglumine was then administered at 1.5 ml per second for post-contrast acquisition.

(III) Determination of Pre-treatment Plasma EBV DNA Titers

Four milliliters (ml) of peripheral blood were drawn and placed in an EDTA tube. All samples were immediately stored in a 4 degrees Celsius refrigerator after blood taking from all patients and they were processed for subsequent EBV DNA extraction within 4

hours of blood taking from patients in the single laboratory of our institution. A total of about 400 to 800 microliters of plasma samples were used for DNA extraction by a QIAamp Blood Kit (Qiagen, Hilden, Germany). The exact amount of plasma was determined for calculation of EBV DNA genome copies. Circulating EBV DNA concentrations were measured using a real-time quantitative polymerase chain reaction (PCR) system with ABI Prism® 7000 Sequence Detection System (Applied Biosystems, USA) that amplified a DNA segment in the *Bam*HI-W fragment region of the EBV genome. All plasma DNA samples were also subject to real-time PCR analysis for the β-*globin* gene, which gave a positive signal on all tested samples. Multiple controls without templates were also included in each analysis as negative controls. All samples were repeated twice on the same day by the same assay for accurate quantification and the results showed that the discrepancy was less than 2% for all repeated samples. All results were expressed as EBV DNA genome copies per ml with accuracy to the nearest 0.1 copy/ml. The lowest detection limit of our plasma EBV DNA assay is 0 copy/ml. Undetectable plasma EBV DNA meant 0 copy/ml and they were used interchangeably in the main text and the Supplementary Materials.

(IV) IMRT Planning Protocol

Every patient was immobilized in the supine position during 18F-FDG PET-CT acquisition and actual treatment by using a thermoplastic head and neck cast. A customised mouthguard was fabricated for better immobilization. MRI images mentioned above were co-registered with the planning PET-CT images for dedicated delineation of the target volumes and organs-at-risk (OARs). OARs, including brainstem, spinal cord, globes, optic nerves, optic chiasm, lenses, temporomandibular joints, temporal lobes, auditory nerves, cochleae, mandible, oral cavity, larynx, parotid glands and vestibules were first contoured. Then gross tumor volumes (GTV) of both the primary tumor and the radiologically involved cervical nodes were outlined. Subsequently, the clinical target volume (CTV-1) for the microscopic disease spread and planning target volume containing CTV-1 with a 3-mm margin (PTV-1) to take into account physiological body motion and set-up errors were generated respectively. Another CTV-2 encompassing the high-risk areas including the posterior half of the maxillary sinuses, nasal cavities, parapharyngeal spaces, styloid processes, basiocciput, basisphenoid, clivus, foramina rotunda and ovale, pterygopalatine fossae, pterygomaxillary fissures, infraorbital fissures, cavernous sinuses, and level Ib and V nodal stations were also outlined subsequently. A corresponding PTV-2 with a 3-mm margin encompassing the CTV-2 was created by Boolean operations of the treatment planning system (Eclipse version 8.0 to 13.0 software, Eclipse Treatment Planning System, Palo Alto, CA, USA), which was also used for IMRT planning using Analytical Anisotropic Algorithm. All the targets and OARs delineation were approved by senior radiation oncologists (Victor Ho-Fun Lee, Dora Lai-Wan Kwong and To-Wai Leung) before dose optimisation for IMRT. During optimization, the maximum dose to the brainstem, optic nerves, and chiasm was limited to 54 Gy and less than 45 Gy to the spinal cord. Allowance was given for some locally advanced tumors in which the maximum dose to the brainstem, optic nerves and chiasm could be up to 60 Gy. Efforts were also made to limit the mean dose to the parotid glands to 26 Gy and the dose to the lenses and temporal lobes to as low as could reasonably be achieved without compromising dose coverage to the PTVs. A dose of 60-74 Gy was prescribed to the PTV-70 and 54-60 Gy to the PTV-2 in 30-35 fractions delivered by simultaneous accelerated radiation therapy technique (SMART). All IMRT planning, dose optimization and quality assurance was performed by a certified medical physicist (Chor-Yi Ng) and all IMRT plans fulfilled acceptance criteria with at least 95% of PTVs having received the prescribed dose, the maximum dose of PTVs limited to 107% or below and the maximum dose of organs-at-risk within tolerance limits according to International Commission on Radiation Units and Measurements (ICRU) criteria. They were then approved by senior radiation oncologists (Victor Ho-Fun Lee, Dora Lai-Wan Kwong and To-Wai Leung) before IMRT commencement. Positional

verification with on-board imaging was performed before and then daily before the first 3 fractions of IMRT followed by weekly afterwards during the whole course of IMRT, to track any anteroposterior and lateral body displacements.

(V) Recursive Partitioning Analysis (RPA)

We performed the first run of recursive partitioning analysis (RPA) with "co-existing liver-bone metastases" identified from univariable (UVA) and multivariable analyses (MVA) to derive a new set of RPA M1 subgroups objectively, with overall survival (OS) as the survival endpoint. The first run of RPA revealed that those with no liver and bone metastases performed better with a significantly longer survival when compared to those with co-existing liver and bone metastases ($p = 0.023$) (Supplementary Figure 1). Internal validation with 1000 bootstrapping replications also confirmed this result. As a result, Set 1, termed as Anatomic-RPA groups, was formed which subdivided M1 into M1a (no liver and bone metastasis) with 3-year OS of 41.8% and M1B (co-existing liver and bone metastases) with 3-year OS of 0% (HR = 2.37, $p = 0.023$).

We performed the second run of RPA with the addition of pretreatment plasma EBV DNA as the only prognostic factor of OS as revealed in UVA and MVA. The RPA of this run found that pretreatment plasma EBV DNA with a cutoff of 2328 copies/ml was an independent poor prognostic group as compared to those with >2328 copies/ml (Supplementary Figure 2A). Since 2328 copies/ml was close to 2500 copies/ml which sounds more integral and representative, we put 2500 copies/ml into RPA again and found that the final result remained the same and 2500 copies/ml remained as a robust cutoff after 1000 bootstrapping replications as internal validation in our statistical analysis (Supplementary Figure 2B). As a result, Set 2, termed as Prognostic-RPA groups, was formed which subdivided M1 significantly into M1a (>2500 copies/ml) with 3-year OS of 74.4% and M1b (≤ 2500 copies/ml) with 3-year OS of 17.1% (HR = 4.38; $p < 0.001$).

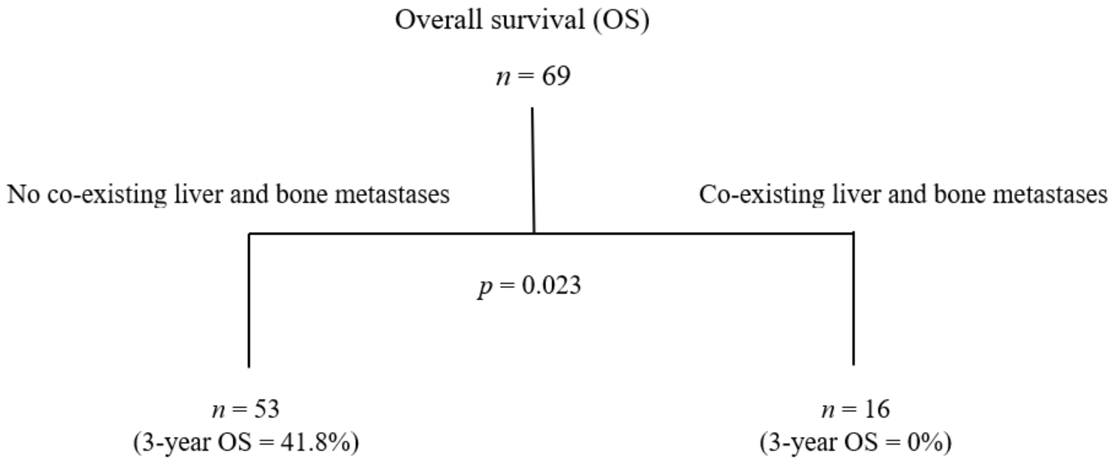

Supplementary Figure S1. The results of recursive partitioning analyses (Set 1) incorporating "Co-existing liver-bone metastases" as the only prognostic factor of OS revealed in univariable analysis and multivariable analysis for the training cohort revealed that M1 categories can be significantly segregated into two groups namely M1a – no co-existing liver-bone metastases, and M1b – co-existing liver-bone metastases.

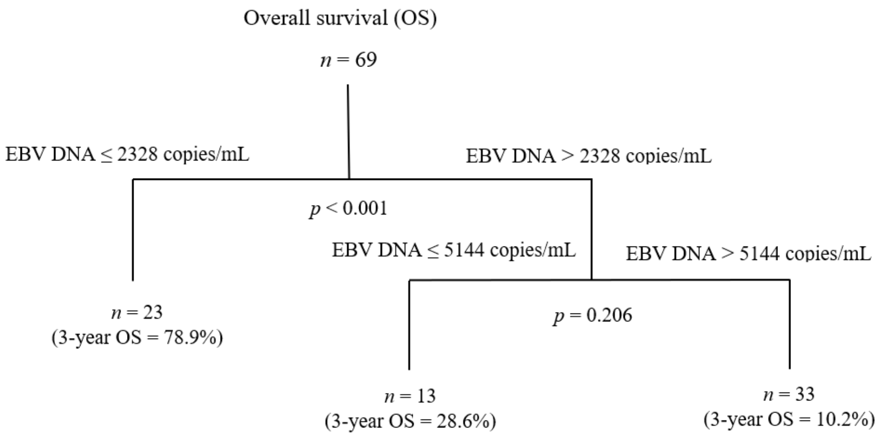

(**a**)

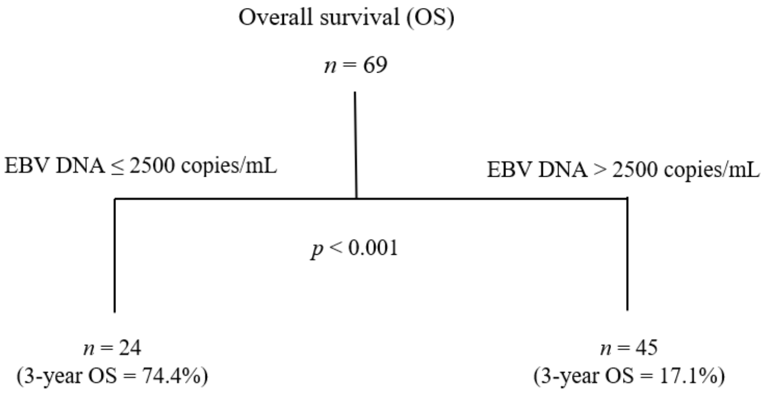

(**b**)

Supplementary Figure S2. The results of recursive partitioning analyses incorporating pretreatment plasma EBV DNA and metastatic characteristics (number of metastasis and site of metastasis) showing (**a**) after the first run which revealed that pretreatment plasma EBV DNA titer of 2328 copies/ml was a significant segregator and (**b**) after the second run by setting 2500 copies/ml as the cutoff which was also confirmed as a significant segregator.

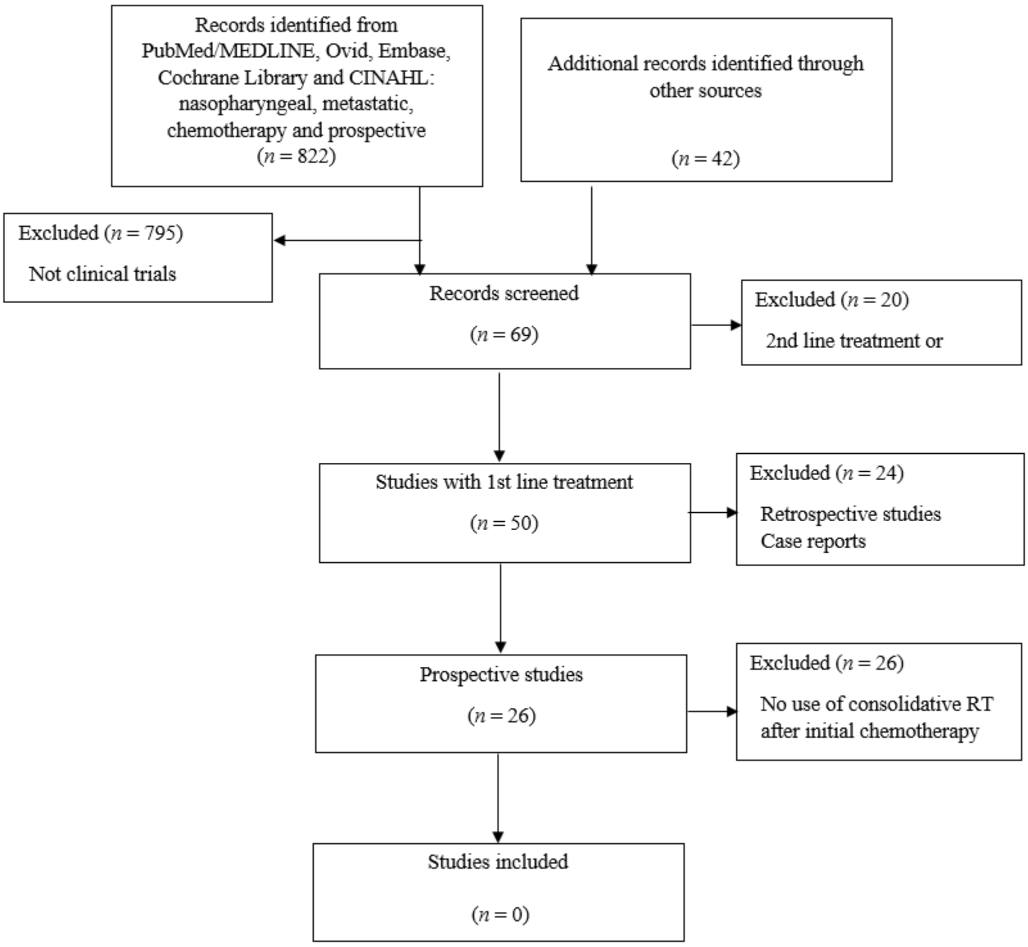

Supplementary Figure S3. Flowchart showing the results of systematic review identified from PubMed/MEDLINE, Ovid, Embase, Cochrane Library and CINAHL.

Supplementary Table S1. Results and details of systemic review on articles for metastatic NPC treated with chemotherapy as initial systemic treatment [1–68].

Reference	Study	Setting	Phase	Disease status	Number of patients	Use of consolidation RT after initial chemotherapy	Use of IMRT as consolidative treatment after 1st line chemotherapy	Use of further RT for metastatic sites	Plasma EBV DNA measurement at the time of metastasis
1	Boussen, et al 1991	1st line or beyond	II	M+R+D	49	N	N	N	N
2	Wang, et al 1991	UNK	Retrospective	M	25	N	N	N	N
3	Dugan, et al 1993	2nd line or beyond	II	M+R+D	108	N	N	N	N
4	Su, et al 1993	1st line	II	M+R+D	25	N	N	N	N
5	Au, et al 1994	1st line	II	R+D	24	N	N	N	N
6	Chi, et al 1994	2nd line or beyond	II	R+D	35	N	N	N	N
7	Azli, et al 1995	1st line or beyond	II	M+R+D	44	N	N	N	N

Reference	Study	Setting	Phase	Disease status	Number of patients	Use of consolidation RT after initial chemotherapy	Use of IMRT as consolidative treatment after 1st line chemotherapy	Use of further RT for metastatic sites	Plasma EBV DNA measurement at the time of metastasis
8	Stein, et al 1996	1st line	II	M+R+D	18	N	N	N	N
9	Yeo, et al 1996	1st line or beyond	II	M	42	N	N	N	N
10	Au, et al 1998	2nd line or beyond	II	M	24	N	N	N	N
11	Siu, et al 1998	1st line	I/II	M+R+D	61	N	N	N	N
12	Yeo, et al 1998	1st line or beyond	II	M+R+D	27	N	N	N	N
13	Hasbini, et al 1999	1st line	II	R+D	44	N	N	N	N
14	Taamma, et al 1999	1st line	II	R+D	23	N	N	N	N
15	Tan, et al 1999	1st line	II	M	32	N	N	N	N
16	Foo, et al 2002	1st line or beyond	II	M	42	N	N	N	N
17	Huang, et al 2002	1st line or beyond	II	R+D	34	N	N	N	N
18	Ma, et al 2002	1st line or beyond	II	M+R+D	32	N	N	N	N
19	McCarthy et al 2002	1st line	II	M+R+D	9	N	N	N	N
20	Ngan, et al 2002	1st line or beyond	II	R+D	44	N	N	N	N
21	Chua, et al 2003	2nd line or beyond	Retrospective	R+D	49	N	N	N	N
22	Altundag, et al 2002	2nd line or beyond	II	R+D	21	N	N	N	N
23	Ciuleanu, et al 2004	1st line	II	M+R+D	40	N	N	N	N
24	Poon, et al 2005	2nd line or beyond	II	M	28	N	N	N	N
25	Wang, et al 2006	2nd line or beyond	II	R+D	39	N	N	N	N
26	Yeh, et al 2006	1st line	Retrospective	M	125	Y	N	N	N
27	Chua, et al 2008	2nd line	II	R+D	17	N	N	N	N
28	Ciuleanu, et al 2008	2nd line or beyond	II	R+D	26	N	N	N	N
29	Leong, et al 2008	1st line	II	M	28	N	N	N	N
30	Li, et al 2008	1st line	II	M	48	N	N	N	N
31	Wang, et al 2008	1st line or beyond	Retrospective	R+D	75	N	N	N	N
32	Zhang, et al 2008	2nd line or beyond	II	R+D	32	N	N	N	N
33	Ma, et al 2009	1st line	II	R+D	40	N	N	N	N
34	Ngeow, et al 2011	2nd line or beyond	II	R+D	30	N	N	N	N

Reference	Study	Setting	Phase	Disease status	Number of patients	Use of consolidation RT after initial chemotherapy	Use of IMRT as consolidative treatment after 1st line chemotherapy	Use of further RT for metastatic sites	Plasma EBV DNA measurement at the time of metastasis
35	Chen, et al 2012	2nd line or beyond	II	R+D	61	N	N	N	N
36	Chua, et al 2012	1st line	II	R+D	19	N	N	N	N
37	Dede, et al 2012	2nd line or beyond	Retrospective	R+D	30	N	N	N	N
38	Ji, et al 2012	1st line	II	M+R+D	47	N	N	N	N
39	Lin, et al 2012	1st line	Retrospective	M	105	Y	N	N	N
40	Setton, et al 2012	1st line	Case reports	M	5	Y	N	N	N
41	Yau, et al 2012	1st line or beyond	II	R+D	15	N	N	N	N
42	Chen, et al 2013	2nd line or beyond	II	M+R+D	95	N	N	N	Y
43	Chen, et al 2013	1st line	Retrospective	M	408	Y	N	N	N
44	Hsieh, et al 2013	2nd line or beyond	II	M+R+D	22	N	N	N	N
45	Lin, et al 2013	1st line	Retrospective	M	226	Y	N	N	N
46	Tsao, et al 2013	2nd line	II	M	13	N	N	N	N
47	Xue, et al 2013	1st line	II	M+R+D	54	N	N	N	N
48	Zeng et al 2014	1st line	Retrospective	M	234	Y	Y	Y	N
49	Hsieh et al 2015	1st line or beyond	II	M+R+D	52	N	N	N	N
50	Hu et al 2015	1st line	Retrospective	M	41	Y	Y	N	N
51	Hu et al 2015	1st line	Retrospective	M	679	Y	N	N	N
52	Zhang et al 2016	1st line or beyond	III	M+R+D	362	N	N	N	N
53	Lee et al 2017	3rd line or beyond	II	R+D	56	N	N	N	Y
54	Peng et al 2017	3rd line or beyond	II	M+R+D	52	N	N	N	N
55	Rusthoven et al 2017	1st line	Retrospective	M	718	Y	Y	N	N
56	Verma et al 2017	1st line	Retrospective	M	555	Y	N	N	N
57	Yin et al 2017	1st line	Retrospective	M	32	Y	Partial (59%)	Partial (31%)	N
58	Zou, et al 2017	1st line or beyond	Retrospective	M	462	Y	Y	N	N
59	Liang et al 2019	2nd line or beyond	Retrospective	R+D	448	N	N	N	N
60	Shuang et al, 2019	2nd line or beyond	Retrospective	M	39	Y	N	N	Y
61	Huang et al, 2020	1st line	Retrospective	M	821	Y	Partial (89%)	Partial (19%)	Y

Reference	Study	Setting	Phase	Disease status	Number of patients	Use of consolidation RT after initial chemotherapy	Use of IMRT as consolidative treatment after 1st line chemotherapy	Use of further RT for metastatic sites	Plasma EBV DNA measurement at the time of metastasis
62	Sun et al, 2020	1st line	Retrospective	M	502	Y	Partial (74%)	N	N
63	Sun et al 2020	1st line or beyond	Retrospective	M+D	266	N	N	N	N
64	Toumi, et al 2020	1st line or beyond	Retrospective	M+D	112	Y	N	N	N
65	Ueda et al 2020	1st line or beyond	Retrospective	M+D	14	N	N	N	N
66	You et al, 2020	1st line	III	M	126	Y	Y	Y	N
67	Zhang et al, 2020	1st line or beyond	II	M+D	43	Y	Y	N	N
68	Zheng et al 2020	1st line or beyond	Retrospective	M+D	817	Y	Y	N	Y

M–de novo metastasis, R–locoregional recurrence, D–distant relapse after prior definitive treatment, Y–yes, N–no, UNK–unknown.

References

1. Boussen, H.; Cvitkovic, E.; Wendling, J.L.; Azli, N.; Bachouchi, M.; Mahjoubi, R.; Kalifa, C.; Wibault, P.; Schwaab, G.; Armand, J.P. Chemotherapy of metastatic and/or recurrent undifferentiated nasopharyngeal carcinoma with cisplatin, bleomycin, and fluorouracil. *J Clin Oncol* **1991**, *9*, 1675–1681.
2. Wang, T.L.; Tan, Y.O. Cisplatin and 5-fluorouracil continuous infusion for metastatic nasopharyngeal carcinoma. *Ann Acad Med Singap.* **1991,** *20*, 601–603.
3. Dugan, M.; Choy, D.; Ngai, A.; Sham, J.; Choi, P.; Shiu, W.; Leung, T.; Teo, P.; Prasad, U.; Lee, S.; et al. Multicenter phase II trial of mitoxantrone in patients with advanced nasopharyngeal carcinoma in Southeast Asia: an Asian-Oceanian Clinical Oncology Association Group study. *J Clin Oncol.* **1993**, *11*, 70–76.
4. Su, W.C.; Chen, T.Y.; Kao, R.H.; Tsao ,C.J. Chemotherapy with cisplatin and continuous infusion of 5-fluorouracil and bleomycin for recurrent and metastatic nasopharyngeal carcinoma in Taiwan. *Oncology* **1993**, *50*, 205–208.
5. Au, E.; Ang, P.T. A phase II trial of 5-fluorouracil and cisplatinum in recurrent or metastatic nasopharyngeal carcinoma. *Ann Oncol*. **1994**, *5*, 87–89.
6. Chi, K.H.; Chan, W.K.; Cooper, D.L.; Yen, S.H.; Lin, C.Z.; Chen, K.Y. A phase II study of outpatient chemotherapy with cisplatin, 5-fluorouracil, and leucovorin in nasopharyngeal carcinoma. *Cancer* **1994**, *15*, 247–252.
7. Azli, N.; Fandi, A.; Bachouchi, M.; Rahal, M.; Lianes, P.; Wibault, P.; Boussen, H.; Eschwege, F.; Armand, J.P.; Cvitkovic, E.; et al. Final report of a phase II study of chemotherapy with bleomycin, epirubicin, and cisplatin for locally advanced and metastatic/recurrent undifferentiated carcinoma of the nasopharyngeal type. *Cancer J Sci Am.* **1995**, *1*, 222–229.
8. Stein, M.E.; Ruff, P.; Weaving, A.; Fried, J.; Bezwoda, W.R.. A phase II study of cisplatin/ifosfamide in recurrent/metastatic undifferentiated nasopharyngeal carcinoma among young blacks in southern Africa. *Am J Clin Oncol.* **1996**, *19*, 386–388.
9. Yeo, W.; Leung, T.W.; Leung, S.F.; Teo, P.M.; Chan, A.T.; Lee, W.Y.; Johnson, P.J. Phase II study of the combination of carboplatin and 5-fluorouracil in metastatic nasopharyngeal carcinoma. *Cancer Chemother Pharmacol*. **1996**, *38*, 466–470.
10. Au, E.; Tan, E.H.; Ang, P.T. Activity of paclitaxel by three-hour infusion in Asian patients with metastatic undifferentiated nasopharyngeal cancer. *Ann Oncol.* **1998**, *9*, 327–329.
11. Siu, L.L.; Czaykowski, P.M.; Tannock, I.F. Phase I/II study of the CAPABLE regimen for patients with poorly differentiated carcinoma of the nasopharynx. *J Clin Oncol.* **1998**, *16*, 2514–2521.
12. Yeo, W.; Leung, T.W.; Chan, A.T.; Chiu, S.K.; Yu, P.; Mok, T.S., Johnson, P.J.. A phase II study of combination paclitaxel and carboplatin in advanced nasopharyngeal carcinoma. *Eur J Cancer.* **1998**, *34*, 2027–2031.
13. Hasbini, A.; Mahjoubi, R.; Fandi, A.; Chouaki, N.; Taamma, A.; Lianes, P.; Cortès-Funes, H.; Alonso, S.; Armand, J.P.; Cvitkovic, E; et al. Phase II trial combining mitomycin with 5-fluorouracil, epirubicin, and cisplatin in recurrent and metastatic undifferentiated carcinoma of nasopharyngeal type. *Ann Oncol*. **1999**, *10*, 421–425.
14. Taamma, A.; Fandi, A.; Azli, N.; Wibault, P.; Chouaki, N.; Hasbini, A.; Couteau, C.; Armand, J.P.; Cvitkovic, E. Phase II trial of chemotherapy with 5-fluorouracil, bleomycin, epirubicin, and cisplatin for patients with locally advanced, metastatic, or recurrent undifferentiated carcinoma of the nasopharyngeal type. *Cancer* **1999**, *1*, 86:1101–1108.
15. Tan, E.H.; Khoo, K.S.; Wee, J.; Fong, K.W.; Lee, K.S.; Lee, K.M.; Chua, E.T.; Tan, T.; Khoo-Tan, H.S.; Yang, T.L.; et al. Phase II trial of a paclitaxel and carboplatin combination in Asian patients with metastatic nasopharyngeal carcinoma. *Ann Oncol.* **1999**, *10*, 235–237.

16. Foo, K.F.; Tan, E.H.; Leong, S.S.; Wee, J.T.; Tan, T.; Fong, K.W.; Koh, L.; Tai, B.C.; Lian, L.G.; Machin, D.; et al. Gemcitabine in metastatic nasopharyngeal carcinoma of the undifferentiated type. *Ann Oncol.* **2002**, *13*, 150–156.
17. Huang, H.Q.; Zhou, Z.M.; Li, Y.H.; Jiang, W.Q.; He, Y.J.; Teng, X.Y.; Guan, X.X. Preliminary results of ifosfamide and doxorubicin regimen in treatment of patients with recurrent and metastatic nasopharyngeal carcinoma. *Ai Zheng*. **2002**, *21*, 409–411.
18. Ma, B.B.; Tannock, I.F.; Pond, G.R.; Edmonds, M.R.; Siu, L.L. Chemotherapy with gemcitabine-containing regimens for locally recurrent or metastatic nasopharyngeal carcinoma. *Cancer* **2002**, *95*, 2516–2523.
19. McCarthy, J.S.; Tannock, I.F.; Degendorfer, P.; Panzarella, T.; Furlan, M.; Siu, L.L. A Phase II trial of docetaxel and cisplatin in patients with recurrent or metastatic nasopharyngeal carcinoma. *Oral Oncol* **2002**, *38*, 686–690.
20. Ngan, R.K.; Yiu, H.H.; Lau, W.H.; Yau, S.; Cheung, F.Y.; Chan, T.M.; Jin, F.; Shi, M.; Chen, Y.P.; Hu, W.H.; et al. Combination gemcitabine and cisplatin chemotherapy for metastatic or recurrent nasopharyngeal carcinoma: report of a phase II study. *Ann Oncol.* **2002**, *13*, 1252–1258.
21. Chua, D.T.; Sham, J.S. ; Au, G.K. A phase II study of capecitabine in patients with recurrent and metastatic nasopharyngeal carcinoma pretreated with platinum-based chemotherapy. *Oral Oncol.* **2003**, *39*, 361–366.
22. Altundag, K.; Aksoy, S.; Gullu, I.; Altundag, O.; Ozyar, E.; Yalcin, S.; Cengiz, M.; Akyol, F. Salvage ifosfamide-doxorubicin chemotherapy in patients with recurrent nasopharyngeal carcinoma pretreated with Cisplatin-based chemotherapy. *Med Oncol.* **2004,** *21*, 211–215.
23. Ciuleanu, T.E.; Fountzilas, G.; Ciuleanu, E.; Plataniotis, M.; Todor, N.; Ghilezan, N. Paclitaxel and carboplatin in relapsed or metastatic nasopharyngeal carcinoma: a multicenter phase II study. *J BUON*. **2004**, *9*, 161–165.
24. Poon, D.; Chowbay, B.; Cheung, Y.B.; Leong, S.S.; Tan, E.H. Phase II study of irinotecan (CPT-11) as salvage therapy for advanced nasopharyngeal carcinoma. *Cancer* **2005**, *103*, 576–581.
25. Wang, C.C.; Chang, J.Y.; Liu, T.W.; Lin, C.Y.; Yu, Y.C.; Hong, R.L. Phase II study of gemcitabine plus vinorelbine in the treatment of cisplatin-resistant nasopharyngeal carcinoma. Head Neck. **2006**, *28*, 74–80.
26. Yeh, S.A.; Tang, Y.; Lui, C.C.; Huang, E.Y. Treatment outcomes of patients with AJCC stage IVC nasopharyngeal carcinoma: benefits of primary radiotherapy. *Jpn J Clin Oncol.* **2006**, *36*, 132–136.
27. Chua, D.; Wei, W.I.; Sham, J.S.; Au, G.K.. Capecitabine monotherapy for recurrent and metastatic nasopharyngeal cancer. *Jpn J Clin Oncol* **2008**, *38*, 244–249.
28. Ciuleanu, E.; Irimie, A.; Ciuleanu, T.E.; Popita, V.; Todor, N.; Ghilezan, N. Capecitabine as salvage treatment in relapsed nasopharyngeal carcinoma: a phase II study. *J BUON*. **2008**, *13*, 37–42.
29. Leong, S.S.; Wee, J.; Rajan, S.; Toh, C.K.; Lim, W.T.; Hee, S.W.; Tay, M.H.; Poon, D.; Tan, E.H. Triplet combination of gemcitabine, paclitaxel, and carboplatin followed by maintenance 5-fluorouracil and folinic acid in patients with metastatic nasopharyngeal carcinoma. *Cancer* **2008**, *113*, 1332–1337.
30. Li, Y.H.; Wang, F.H.; Jiang, W.Q.; Xiang, X.J.; Deng, Y.M.; Hu, G.Q.; Xu, D.M.; Chen, Y.; Lin, Q.; He, Y.J. Phase II study of capecitabine and cisplatin combination as first-line chemotherapy in Chinese patients with metastatic nasopharyngeal carcinoma. *Cancer Chemother Pharmacol*. **2008**, *62*, 539–544.
31. Wang, J.; Li, J.; Hong, X.; Tang, W.; Hu, X.; Guo, Y. Retrospective case series of gemcitabine plus cisplatin in the treatment of recurrent and metastatic nasopharyngeal carcinoma. *Oral Oncol.* **2008**, *44*, 464–470.
32. Zhang, L.; Zhang, Y.; Huang, P.Y.; Xu, F.; Peng, P.J.; Guan, Z.Z. Phase II clinical study of gemcitabine in the treatment of patients with advanced nasopharyngeal carcinoma after the failure of platinum-based chemotherapy. *Cancer Chemother Pharmacol*. **2008** *61*, 33–38.
33. Ma, B.B.; Hui, E.P.; Wong, S.C.; Tung, S.Y.; Yuen, K.K.; King, A.; Chan, S.L.; Leung, S.F.; Kam, M.K.; Yu, B.K.; et al. Multicenter phase II study of gemcitabine and oxaliplatin in advanced nasopharyngeal carcinoma--correlation with excision repair cross-complementing-1 polymorphisms. *Ann Oncol*. **2009**, *20*, 1854–1859.
34. Ngeow, J.; Lim, W.T.; Leong, S.S.; Ang, M.K.; Toh, C.K.; Gao, F.; Chowbay, B.; Tan, E.H. Docetaxel is effective in heavily pretreated patients with disseminated nasopharyngeal carcinoma. *Ann Oncol*. **2011,** *22*, 718–722.
35. Chen, C.; Wang, F.H., Wang, Z.Q.; An, X.; Luo, H.Y.; Zhang, L.; Chen, Y.C.; Xu, R.H.; Li, Y.H. Salvage gemcitabine-vinorelbine chemotherapy in patients with metastatic nasopharyngeal carcinoma pretreated with platinum-based chemotherapy. *Oral Oncol*. **2012**, *48*, 1146–1151.
36. Chua, D.T.; Yiu, H.H.; Seetalarom, K.; Ng, A.W.; Kurnianda, J.; Shotelersuk, K.; Krishnan, G.; Hong, R.L.; Yang, M.H.; Wang, C.H.; et al. Phase II trial of capecitabine plus cisplatin as first-line therapy in patients with metastatic nasopharyngeal cancer. *Head Neck.* **2012**, *34*, 1225–1230.
37. Dede, D.S.; Aksoy, S.; Cengiz, M.; Gullu, I.; Altundag, K. Ifosfamide and doxorubicin combination chemotherapy for recurrent nasopharyngeal carcinoma patients. *Asian Pac J Cancer Prev*. **2012**, *13*, 2225–2228.
38. Ji, J.H.; Korean Cancer Study Group (KCSG), Yun, T.; Kim, S.B.; Kang, J.H.; Park, J.C.; Cho, I.S.; Sohn, C.H.; Heo, D.S.; Jang, J.S.; et al. A prospective multicentre phase II study of cisplatin and weekly docetaxel as first-line treatment for recurrent or metastatic nasopharyngeal cancer (KCSG HN07-01). *Eur J Cancer*. **2012**, *48*, 3198–3204.
39. Lin, S.; Tham, I.W.; Pan, J.; Han, L.; Chen, Q.; Lu, J.J. Combined high-dose radiation therapy and systemic chemotherapy improves survival in patients with newly diagnosed metastatic nasopharyngeal cancer. *Am. J. Clin. Oncol.* **2012**, *35*, 474–479.
40. Setton, J.; Wolden, S.; Caria, N.; Lee, N. Definitive treatment of metastatic nasopharyngeal carcinoma: Report of 5 cases with review of literature. *Head Neck.* **2012**, *34*, 753–757.

41. Yau, T.K.; Shum, T.; Lee, A.W.; Yeung, M.W.; Ng, W.T.; Chan, L. A phase II study of pemetrexed combined with cisplatin in patients with recurrent or metastatic nanopharyngeal carcinoma. *Oral Oncol.* **2012**, *48*, 441–444.
42. Chen, C.; Wang, F.; Wang, Z.; Li, C.; Luo, H.; Liang, Y.; An, X.; Shao, J.; Li, Y.; et al. Polymorphisms in ERCC1 C8092A predict progression-free survival in metastatic/recurrent nasopharyngeal carcinoma treated with cisplatin-based chemotherapy. *Cancer Chemother Pharmacol.* **2013**, *72*, 315–322.
43. Chen, M.-Y.; Jiang, R.; Guo, L.; Zou, X.; Liu, Q.; Sun, R.; Qiu, F.; Xia, Z.-J.; Huang, H.-Q.; Zhang, L.; et al. Locoregional radiotherapy in patients with distant metastases of nasopharyngeal carcinoma at diagnosis. *Chin. J. Cancer* **2013**, *32*, 604–613, https://doi.org/10.5732/cjc.013.10148.
44. Hsieh, C.H.; Hsu, C.L.; Wang, C.H.; Liaw, C.C.; Chen, J.S.; Chang, H.K.; Yang, T..S; Chang, J.W.; Lin, Y.C.; Liau, C.T.; et al. Cisplatin, tegafur-uracil and leucovorin plus mitomycin C: an acceptably effective and toxic regimen for patients with recurrent or metastatic nasopharyngeal carcinoma. *Biomed J.* **2013**, *36*, 229–236.
45. Lin, H. ; Jin, H.X. ; Cai, X.Y.; Guo, L. ; Wang, H. ; Hu, Wh. ; Sun, Y. Chemotherapy plus radiotherapy makes curability a possibility in nasopharyngeal carcinoma patients with distant metastasis at diagnosis. *Head Neck Oncol* **2013**, 5.
46. Tsao, A.; Hui, E.P.; Juergens, R.; Marur, S.; Huat, T.E.; Cher, G.B.; Hong, R.L.; Hong, W,K,; Chan, A.T. Phase II study of TAS-106 in patients with platinum-failure recurrent or metastatic head and neck cancer and nasopharyngeal cancer. *Cancer Med.* **2013**, *2*, 351–359.
47. Xue, C.; Huang, Y. ; Huang, P.Y. ; Yu, Q.T.; Pan, J.J. ; Liu, L.Z. ; Song, X.Q. ; Lin, S.J. ; Wu, J.X. ; Zhang, J.W. ; et al. Phase II study of sorafenib in combination with cisplatin and 5-fluorouracil to treat recurrent or metastatic nasopharyngeal carcinoma. *Ann Oncol.* **2013**, *24*, 1055–1061.
48. Zeng, L.,; Tian, Y.M.; Huang, Y.; Sun, X.M.; Wang, F.H.; Deng, X.W.; Han, F.; Lu, T.X. Retrospective analysis of 234 nasopharyngeal carcinoma patients with distant metastasis at initial diagnosis: therapeutic approaches and prognostic factors. *PLoS One*. **2014**, 23:e108070.
49. Hsieh, J.C.; Hsu, C.L.; Ng, S.H.; Wang, C.H.; Lee, K.D.; Lu, Chet.; Chang, Y.F.; Hsieh, R.K.,; Yeh, K.H.; Hsiao, C.H.; et al. Gemcitabine plus cisplatin for patients with recurrent or metastatic nasopharyngeal carcinoma in Taiwan: a multicenter prospective Phase II trial. *Jpn J Clin Oncol*. **2015,** 45, 819–827.
50. Hu, S.X.; He, X.H.; Dong, M.; Jia, B.; Zhou, S.Y.; Yang, J.L.; Yang, S.; Zhang, C.G.; Liu, P.; Qin, Y.; et al. Systemic chemotherapy followed by locoregional definitive intensity-modulated radiation therapy yields prolonged survival in nasopharyngeal carcinoma patients with distant metastasis at initial diagnosis. *Med Oncol.* **2015**, *32*, 224.
51. Hu, J.; Kong, L.; Gao, J.; Hu, W.; Guan, X.; Lu, J.J. Use of Radiation Therapy in Metastatic Nasopharyngeal Cancer Improves Survival: A SEER Analysis. *Sci Rep.* 2017, *7*, 721.
52. Zhang, L.; Huang, Y.; Hong, S.; Yang, Y.; Yu, G.; Jia, J.; Peng, P.; Wu, X.; Lin, Q.; Xi, X.; et al. Gemcitabine plus cisplatin versus fluorouracil plus cisplatin in recurrent or metastatic nasopharyngeal carcinoma: a multicentre, randomised, open-label, phase 3 trial. *Lancet* **2016**, *388*, 1883–1892, https://doi.org/10.1016/s0140-6736(16)31388-5.
53. Lee, V.H.F.; Kwong, D.L.W.; Lam, K.O.; Lai, Y.C.; Li, Y.; Tong, C.C.; Ho, P.P.Y.; Chan, W.L; Wong, L.S.; Leung, D.K.C.; et al. Metronomic oral cyclosphosphamide as third-line systemic treatment or beyond in patients with inoperable locoregionally advanced recurrent or metastatic nasopharyngeal carcinoma. *Medicine (Baltimore).* **2017**, 96:e6518
54. Peng, P.J.; Lv, B.J.; Wang, Z.H.; Liao, H.; Liu, Y.M.; Lin, Z.; Con, Y.Y.; Huang, P.Y.; et al. Multi-institutional prospective study of nedaplatin plus S-1 chemotherapy in recurrent and metastatic nasopharyngeal carcinoma patients after failure of platinum-containing regimens. *Ther Adv Med Oncol.* **2017**, *9*, 68–74.
55. Rusthoven, C.G.; Lanning, R.M.; Jones, B.L.; Amini, A.; Koshy, M.; Sher, D.J.; Bowles, D.W.; McDermott, J.D.; Jimeno, A.; Karam, S.D.; et al. Metastatic nasopharyngeal carcinoma: Patterns of care and survival for patients receiving chemotherapy with and without local radiotherapy. *Radiother Oncol.* **2017**, *124*, 139–146.
56. Verma, V.; Allen, P.K.; Simone, C.B.; Gay, H.A.; Lin, S.H. Addition of Definitive Radiotherapy to Chemotherapy in Patients With Newly Diagnosed Metastatic Nasopharyngeal Cancer. *J Natl Compr Canc Netw*. **2017**, *15*, 1383–1391.
57. Yin, Z.; Zhang, X.; Wang, Y.; Wang, P.; Yuan, Z. The combination of systemic therapy and locoregional radiotherapy prolongs survival in newly diagnosed metastatic nasopharyngeal carcinoma patients. *Onco Targets Ther*. **2017**, *27*, 10:5677–5683.
58. Zou, X.; You, R.; Liu, H.; He, Y.-X.; Xie, G.-F.; Xie, Z.-H.; Li, J.-B.; Jiang, R.; Liu, L.-Z.; Li, L.; et al. Establishment and validation of M1 stage subdivisions for de novo metastatic nasopharyngeal carcinoma to better predict prognosis and guide treatment. *Eur. J. Cancer* **2017**, *77*, 117–126, https://doi.org/10.1016/j.ejca.2017.02.029.
59. Liang, Y.J.; Sun, X.S.; Yang, Z.C.; Tang, Q.N.; Guo, S.S.; Liu, L.T.; Xie, H.J.; Liu, S.L.; Yan, J.J.; Li, X.Y.; et al. Effect of local treatment for metastasis and its sequence with chemotherapy on prognosis of post-treatment metastatic nasopharyngeal carcinoma patients. *Oral Oncol*. **2019,** 92, 40–45.
60. Shuang, H.; Feng, J.; Caineng, C.; Qifeng, J.; Tin, J.; Yuanyuan, C.; Xiaozhong, C. The value of radical radiotherapy in the primary tumor of newly diagnosed oligo-metastatic nasopharyngeal carcinoma patients. *Clin. Transl. Oncol.* **2018**, *21*, 213–219, https://doi.org/10.1007/s12094-018-1911-7.
61. Huang, T.; Su, N.; Zhang, X.; Ma, S.; Zhong, G.; Tian, X.; Chen, Q.; Tang, L.; Lu, L.; Fang, Y.; et al. Systemic chemotherapy and sequential locoregional radiotherapy in initially metastatic nasopharyngeal carcinoma: Retrospective analysis with 821 cases. *Head Neck.* **2020,** *42*, 1970–1980.

62. Sun, X.S.; Wang, X.H.; Liu, S.L.; Luo, D.H.; Sun, R.; Liu, L.T.; Guo, S.S.; Chen, Q.Y.; Tang, L.Q.; Mai, H.Q. Comparison of Gemcitabine Plus Cisplatin vs. Docetaxel Plus Fluorouracil Plus Cisplatin Palliative Chemotherapy for Metastatic Nasopharyngeal Carcinoma. *Front Oncol*. **2020,** *10*, 1295.
63. Sun, X.S. ; Liang, Y.J. ; Chen. Q.Y. ; Guo, S.S. ; Liu, L.T. ; Sun, R. ; Luo, D.H. ; Tang, L.Q. ; Mai, H.Q. Optimizing the Treatment Pattern for De Novo Metastatic Nasopharyngeal Carcinoma Patients: A Large-Scale Retrospective Cohort Study. *Front Oncol*. **2020,** *10*, 543646.
64. Toumi, N.; Ennouri, S.; Charfeddine, I.; Daoud, J.; Khanfir, A. Prognostic factors in metastatic nasopharyngeal carcinoma. *Braz J Otorhinolaryngol.* **2020**, *4*, S1808–8694
65. Ueda, Y.; Enokida, T.; Okano, S.; Fujisawa, T.; Ito, K.; Tahara, M. Combination Treatment With Paclitaxel, Carboplatin, and Cetuximab (PCE) as First-Line Treatment in Patients With Recurrent and/or Metastatic Nasopharyngeal Carcinoma. *Front Oncol.* **2020**, *10*, 571304.
66. You, R.; Liu, Y.P.; Huang, P.Y.; Zou, X.; Sun, R.; He, Y.X.; Wu, Y.S.; Wu, Y.S.; Shen, G.P.; Zhang, H.D.; et al. Efficacy and safety of locoregional radiotherapy with chemotherapy vs chemotherapy alone in de novo metastatic nasopharyngeal carcinoma. *JAMA Oncol.* **2020**, *6*, 1345–1352.
67. Zhang, M. ; Huang, H. ; Li, X. ; Huang, Y. ; Chen, C. ; Fang, X. ; Wang, Z. ; Guo, C. ; Lam, S. ; Fu, X. ; et al. Long-Term Survival of Patients With Chemotherapy-Naïve Metastatic Nasopharyngeal Carcinoma Receiving Cetuximab Plus Docetaxel and Cisplatin Regimen. *Front Oncol.* **2020**, *10*, 1011.
68. Zheng, W.H.; He, X.J.; Chen, F.P.; Lin, L.; Huang, X.D.; Zhou, H.Q.; Kou, J.; Lv, J.W.; Ma, J.; Zhou, G.Q.; et al. Establishing M1 stage subdivisions by incorporating radiological features and Epstein-Barr virus DNA for metastatic nasopharyngeal carcinoma. Ann. Transl. *Med.* **2020**, *8*, 83.
